# A quantitative study on growth variability and production of ochratoxin A and its derivatives by *A. carbonarius* and *A. niger* in grape-based medium

**DOI:** 10.1038/s41598-018-32907-z

**Published:** 2018-10-01

**Authors:** Luísa Freire, Tatiane M. Guerreiro, Arthur K. R. Pia, Estela O. Lima, Diogo N. Oliveira, Carlos F. O. R. Melo, Rodrigo R. Catharino, Anderson S. Sant’Ana

**Affiliations:** 1 Department of Food Science, Faculty of Food Engineering, University of Campinas, Campinas, SP Brazil; 2 Innovare Biomarkers Laboratory, Faculty of Pharmaceutical Sciences, University of Campinas, Campinas, SP Brazil

**Keywords:** Applied microbiology, Fungal biology

## Abstract

*Aspergillus carbonarius* and *Aspergillus niger* are the main responsible fungi for the accumulation of ochratoxin A (OTA) in wine grapes. Some strains are able to convert the parent mycotoxin into other compounds by means of hydrolysis and/or conjugation reactions through their defense mechanisms and enzymatic activity, leading to the formation of a modified mycotoxin. Thus, the variability of growth and metabolite production are inherent to the strain, occurring distinctively even when submitted to similar conditions. In this sense, this contribution aimed at determining the variability in multiplication and production of OTA by strains of *A. carbonarius* and *A. niger* isolated from grapes, as well as investigating the formation of modified mycotoxins. Strains were incubated in grape-based medium, and the diameter of the colonies measured daily. The determination of OTA was performed by high-performance liquid chromatography and the identification of modified mycotoxins was carried out using high-resolution mass spectrometry. Variabilities in terms of growth and OTA production were assessed across five different strains. Peak production of OTA was detected on day 15, and a decline on day 21 was observed, indicating that the observed reduction may be associated with the degradation or modification of the OTA over time by the fungus. Ethylamide ochratoxin A, a modified mycotoxin identified in this study, provides evidence that there may be underreporting of total mycotoxin levels in food, increasing uncertainty concerning health risks to the population.

## Introduction

Grapes can be easily contaminated with filamentous fungi in any of the many processes across the production chain. The mycotoxigenic fungi most often associated with this context are from the *Aspergillus* genus, with *A. niger* and *A. carbonarius* figuring as the most relevant species. In addition to deteriorating the food product, these species are also responsible for the production of ochratoxin A (OTA), the main mycotoxin detected in grapes and derivatives (wine and juice)^1,2^. *A. carbonarius* is less common in grapes when compared to *A. niger* species; nonetheless, they are more relevant due to the increased proportion of high-level OTA-producing isolates, which are the ones responsible for the accumulation of OTA in grapes and derivatives^3,4^. OTA is a major clinically relevant mycotoxin, as it may accumulate in the circulatory system, liver, kidneys, and other tissues, such as adipose and muscle, potentially causing immunosuppressive^5^, teratogenic^6^, neurotoxic^7^, genotoxic^8^, mutagenic^9^ and carcinogenic effects^10^.

In addition to the production of secondary metabolites, some *Aspergillus* strains are able to convert the parent mycotoxin into OTA-related compounds through hydrolysis (degradation) and/or conjugation reactions, relying on the fungi’s defense mechanisms and enzymatic complexes^11^. The formed metabolites present alterations in their structure when compared to parent mycotoxin, and are therefore named modified mycotoxins (MMs)^12,13^. Additionally, some strains are also capable of reconverting the modified mycotoxin into the parent mycotoxin, albeit the exact mechanisms are yet to be unraveled^13^. Within this context, it is possible to infer that there may be underreporting of the total levels of mycotoxins in a given sample, as their modified counterparts may not be detected by traditional methods. Moreover, considering that some MMs may be as toxic as the parent species due to structural similarities, there may also be an increase in the total mycotoxin intake through the diet^14^.

The detection of modified mycotoxins remains a major challenge today, as these compounds may contribute to overall toxicity associated with food and have been neglected by legislation. In this scenario, inaccurate risk estimates may result in noneffective risk management measures to protect public health^15^.

Predictions of the fungal growth and metabolite production can be carried out through mathematical models; nonetheless, differences between predicted and actual values are often detected due to intrinsic variability, thereby impacting the Hazard Analysis^16^. Although the variability in the multiplication and production capacity of mycotoxins across different strains of the same fungal species have already been reported in the literature^17–19^, they are yet to be systematically quantified, characterized, and compared. This is relevant to provide more realistic safety margins for both product and process designs, since these parameters may have different impact combined than when evaluated individually^20^. Given that, this study aims at determining the variability of multiplication and OTA production by strains of *A. carbonarius* and *A. niger* isolated from grapes, as well as investigating the formation of modified mycotoxins by these fungi.

## Results and Discussion

### Evaluation of growth and mathematical modeling of strains of *A. carbonarius* e *A. niger*

**Figure 1:**
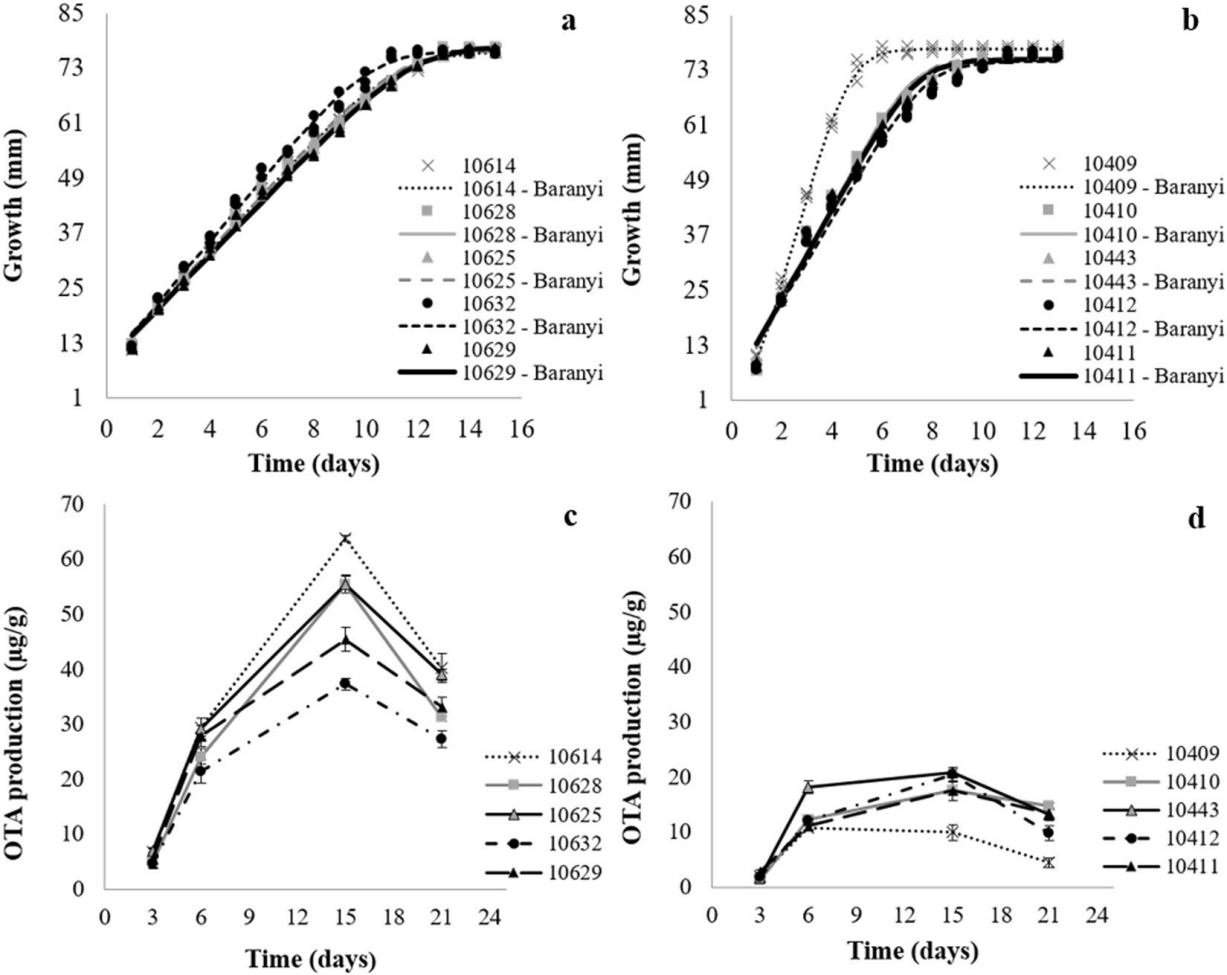
Growth kinetics of *A. carbonarius* strains (10628, 10632, 10614, 10625, 10629) (**a**) and *A. niger* strains (10409, 10410, 10443, 10412, 10411) (**b**) after Baranyi model fitting; and Kinetics of OTA production by *A. carbonarius* (**c**) and *A. niger* (**d**) strains in grape-based culture medium on days 3, 6, 15 and 21.

Growth curves were obtained from diameters of the colonies (mm) vs. time (days) (Fig. 1a,b). The kinetic parameters of growth: growth rate, lag phase and maximum diameter were estimated after the fit of the Baranyi model to the data (Table 1). An excellent Baranyi model fit is observed for both *A. carbonarius* and *A. niger* strains, with low standard deviation, indicating good reproducibility. This model has become the most used and also the most suitable for evaluation of fungi growth due to its good data fit^21–23^.

**Table 1:** Growth parameters of *A. carbonarius* and *A. niger*, obtained after fit the Baranyi model.

Species	Strains	Growth rate (µ; mm.day^−1^)	Lag phase (λ; days)	Maximum diameter (mm)	R^2^
*Aspergillus carbonarius*	10629	5.84^a^ ± 0.14	NA	77.95^d^ ± 0.63	0.99
	10628	6.04^a^ ± 0.17	NA	77.70^d^ ± 0.63	0.99
	10625	6.05^a^ ± 0.20	NA	77.07^cd^ ± 1.49	0.99
	10614	6.05^a^ ± 0.10	NA	76.55^bcd^ ± 0.79	0.99
	10632	6.75ª ± 0.52	NA	77.06^cd^ ± 0.42	0.99
*Aspergillus niger*	10412	9.11^b^ ± 0.20	NA	74.87ª ± 0.44	0.98
	10411	9.74^bc^ ± 0.60	NA	75.27^ab^ ± 0.86	0.98
	10443	10.14^c^ ± 0.54	NA	75.67^abc^ ± 1.06	0.98
	10410	10.23^c^ ± 0.66	NA	75.18^ab^ ± 0.90	0.98
	10409	19.32^d^ ± 1.03	1.12 ± 0.07	77.65^d^ ± 0.73	0.99

*Different letters show statistically significant difference at p < 0.05.

NA: no lag was obtained in this case.

All curves showed an upper asymptote due to the limitation caused by the plate diameter (80 mm), which prevents multiplication, thus generating the maximum diameter (mm) for all strains (between 76.55–77.95 mm for *A carbonarius* and 74.87–77.65 mm for *A. niger*). Most strains of *A. niger* reached the maximum diameter (mm) before the strains of *A. carbonarius*, taking 8 to 13 days and 12 to 15 days of incubation, respectively.

Normally, under unrestricted growth conditions, fungal growth is described through a lag phase and a linear phase, and in some cases the lag phase is not observed. The stationary phase (upper asymptote) is observed only when the fungus is under sub-optimal conditions, such as unfavorable water activity, temperature or pH^24^. This was not the case, as there are no limitations in these factors, only the restriction of growth by reaching the diameter of the Petri dish.

We did not observe lag phase for any of the strains studied after model adjustment, except for strain 10409 of *A. niger*, which presented a lag phase of λ = 1.12 days. We hypothesize that the lag phase of most strains occurs in the 0 to 24-hour period and therefore could not be obtained. Therefore, the sooner the measurements of the colony diameter are performed, the better the precision in determining the lag phase. However, this parameter has no biological significance for fungi, since it is calculated from macroscopic observations of the colony. Despite that, in a study with *Mucor racemosus*, in which the initial inoculum was controlled, lag phase coincided with the end of germination^25^. In addition, lag phase is also dependent on the number of conidia inoculated. The higher the inoculum, the lower the lag phase, so it is of fundamental importance to take into account the size of the inoculum in the model, although this correlation is not observed across inoculum sizes and growth rates^26,27^.

Growth rate values ranged from 5.84–6.75 mm.day^−1^ for *A. carbonarius* strains, but there was no statistical difference between strains (p < 0.05). For *A. niger*, all strains presented a higher comparative growth rate, varying between 9.11–19.32 mm.day^−1^, with strain 10409 presenting the highest growth rate among all others (19.32 mm.day^−1^).

Models developed for bacteria, such as the Baranyi model, can be used to evaluate the kinetics of fungal growth^27^. Furthermore, models generated from experiments carried out in culture medium may also be used to extrapolate the behavior and physiology of the microorganisms in a food to improve quality and safety^27,28^. However, the specificities of these microorganisms must be considered.

### OTA production by *A. carbonarius* and *A. niger* strains

The strains of *A. carbonarius* and *A. niger* were previously evaluated for OTA production capacity by the the Plug Agar method in thin layer chromatography (data not shown). All strains showed the characteristic retention factor and fluorescence spot similar to OTA pattern and, therefore, were considered potentially ochratoxigenic.

In the assay evaluating OTA production over a 21-day incubation in grape-based culture medium, all strains of *A. carbonarius* and *A. niger* were OTA producers on each day of experiment. The lowest levels of OTA were observed at day 3 for all strains. On the other hand, the highest production of this mycotoxin was detected on day 15, when all the strains had already reached the maximum diameter. Thereafter, a reduction in mycotoxin levels was observed (Fig. 1c,d). Lappa, *et al*.^29^ also observed the production of OTA by strains of *A. carbonarius* beginning at the third day of experiment. Maximum levels, nonetheless, were observed between the ninth and eleventh day, although this was the last day of the experiment. Astoreca, *et al*.^30^ also detected differences on the day of maximum production for two strains of *A. niger* aggregate; strain RCP176 had its peak production at day 7 for all conditions, and strain RCP42 at day 14 with 0.995 water activity and a range between 25 and 30 °C. Additionally, strain RCP42 was also considered the largest OTA producer.

In all days of experiments, *A. carbonarius* strains produced the highest levels of OTA when compared to strains of *A. niger*. On day 6, *A. carbonarius* strains: 10614, 10625, and 10629 were the largest producers of OTA, with levels of 29.52, 29.23 and 27.9 μg/g, respectively. On day 15, the highest concentration of OTA was produced only by strain 10614 (*A. carbonarius*), 63.79 μg/g. On day 21, strains 10614 and 10625 (*A. carbonarius*) were the largest producers, 40.32 μg/g and 39.04 μg/g, respectively (Table 2).

**Table 2:** OTA production ( μg/g) by *A. carbonarius* and *A. niger* strains along 21 days of incubation in grape-based culture medium.

Species	Strains	3 (days)	6 (days)	15 (days)	21 (days)
*Aspergillus niger*	10409	2.10^Aa^ ± 0.44	10.89^A^ ± 0.51^c^	9.98^Ac^ ± 1.37	4.49^Ab^ ± 0.82
	10410	1.34^Aa^ ± 0.49	12.24^Ab^ ± 0.72	17.73^Bd^ ± 0.87	14.69 ^Cc^ ± 0.63
	10443	1.73^Aa^ ± 0.29	18.16^Bc^ ± 1.16	20.82 ^Cd^ ± 0.67	13.31^Cb^ ± 1.18
	10412	1.92^Aa^ ± 0.26	12.22^Ac^ ± 0.91	20.52 ^Cd^ ± 1.38	9.87^Bb^ ± 1.35
	10411	2.86^Aba^ ± 0.36	11.14^Ab^ ± 0.67	17.58^Bd^ ± 1.75	13.45 ^Cc^ ± 0.72
*Aspergillus carbonarius*	10628	4.95^Bca^ ± 0.90	24.19^Db^ ± 1.47	55.49^Fd^ ± 1.64	31.29^Ec^ ± 0.80
	10632	4.80^BCa^ ± 0.92	21.46^Cb^ ± 2.07	37.33^Dd^ ± 1.02	27.37^Dc^ ± 1.49
	10614	6.90^Ca^ ± 0.68	29.52^Eb^ ± 1.61	63.79^Gd^ ± 0.58	40.32^Fc^ ± 2.66
	10625	7.02^Ca^ ± 0.43	29.23^Eb^ ± 1.83	55.45^Fd^ ± 1.60	39.04^Fc^ ± 1.02
	10629	5.64^Ca^ ± 1.14	27.90^Eb^ ± 1.82	45.46^Ed^ ± 2.20	33.20^Ec^ ± 1.64

*Mean followed by lowercase letters compare the OTA production by the strain in the line at different times, and uppercase letters compare the OTA production by the strains in the column. Different letters show statistically significant difference at p < 0.05.

Among the filamentous fungi detected in grapes, these species are the most commonly found, accounting for up to 98.5% of fungi of the *Aspergillus* genus isolated from grapes^1^. Although the detection of *A. carbonarius* has a lower incidence in grapes, this species is highly relevant for risk analysis, since it produces higher levels of OTA when compared to *A. niger*^2–4,31^. Moreover, it is possible that strains of the *A. carbonarius* species were adapted to the grape-based culture medium differently than strains of *A. niger*, and therefore, higher levels of OTA were detected. Requirements for OTA production vary across species, and are more restricted when compared to the requirements for their growth^32^.

According to Passamani, *et al*.^31^, both *A. niger* and *A. carbonarius* produced higher levels of OTA in semi-synthetic grape medium under conditions: 15 °C, 0.99 aw and pH 5.35. Optimal growing conditions were: 24 to 37 °C, water activity greater than 0.95 and pH between 4 and 6.5 for *A. niger* and 20 to 33 °C, 0.95 to 0.99 aw and pH between 5 and 6.5 for *A. carbonarius*. Although the optimal conditions for OTA production were the same for both *A. carbonarius* and *A. niger*, the levels of mycotoxin produced by *A. carbonarius* were higher. However, in this study only one strain of each species was used, which does not allow to evaluate the variability between the strains.

Between days 15 and 21, reductions in OTA levels were observed for both *A. carbonarius* and *A. niger*, from 26.67 to 43.61%, and 17.12 to 54.98%, respectively. For *A. carbonarius*, the greatest reduction was observed in strain 10628, and for *A. niger*, the greatest reduction was observed in strain 10409. We hypothesize that such reductions in OTA levels may be related to the formation of modified mycotoxins. OTA, after being produced by the fungus, may either have been transformed into derived metabolites by the action of the enzyme complex of the fungus itself, or became strongly adsorbed by the culture medium matrix over the days of incubation (15 to 21) and was not extracted by the employed method.

Seefelder *et al*.^33^ showed that fumonisins are able to bind to polysaccharides and proteins. Therefore, it is possible that OTA and any related compound may have bound with polysaccharides present in the grape-based culture medium, and were ultimately not detected. Brodehl, *et al*.^11^ indicate that up to 50% of the parent mycotoxin may be strongly adsorbed to the culture medium or fungi mycelium through the formation of adducts and thus not detected. The adsorption of zearalenone in *A. niger* mycelium was also observed as one of the main forms of detoxification of this mycotoxin, in addition to the metabolism of mycotoxin by the fungus generating degraded compounds of lower toxicity^34^. Conidial suspension of *A. niger*, *A. carbonarius* and *A. japonicus* were also able to adsorb OTA, probably through hydrophobic interactions, apart from converting it into α-OTA after germination and growth^35^.

Astoreca, *et al*.^36^, when evaluating OTA production by two strains of *A. niger* aggregate, also observed a reduction in toxin levels on day 21 in almost all trials. This reduction was justified by a possible degradation of the mycotoxin by the strain itself, which would use OTA as an alternative carbon source to continue maintaining its metabolic rate. Lappa, *et al*.^29^ also observed a reduction in OTA levels for some strains of *A. carbonarius* evaluated in culture medium. In studies with orange juice, the reduction in OTA levels, produced by an *A. niger* strain, was observed only on the twenty-eighth day. However, another tested strain showed an intense reduction from the seventh day^37^. Romero, *et al*.^38^ also detected a reduction in OTA levels produced by two strains of *A. carbonarius* on the twenty-eighth day, although for the other two strains this reduction was not observed. Other findings also corroborate this hypothesis, in which *Aspergillus* section *Nigri* strains demonstrate the ability to degrade OTA by generating α-OTA through its enzymatic complex. It is possible that the fungus removes and assimilates phenylalanine from OTA to be used as a source of nitrogen, since nutrients may be scarce at the end of the incubation period^39,40^. Nevertheless, OTA-producing *A. carbonarius* strains were also producing α-OTA^41^, which renders inconclusive the assertion that the formation of α-OTA is only due to the degradation of OTA, and may also be related to the metabolism of the fungus itself.

OTA levels did not correlate with growth rate. Although *A. niger* strains had the highest growth rates, they had lower OTA concentrations when compared to *A. carbonarius* strains. However, among strains of the same species (*A. niger*), a positive correlation was observed: strain 10409 showed the highest growth rate and the highest OTA level on days 15 and 21. According to Astoreca, *et al*.^36^, higher growth rates are associated with lower levels of OTA production, and may even be related to a restriction in the production of this metabolite. In contrast, Lappa, *et al*.^29^ found a positive correlation between growth parameters and OTA production levels.

### Variability of multiplication and OTA production by *A. niger* and *A. carbonarius*

**Figure 2:**
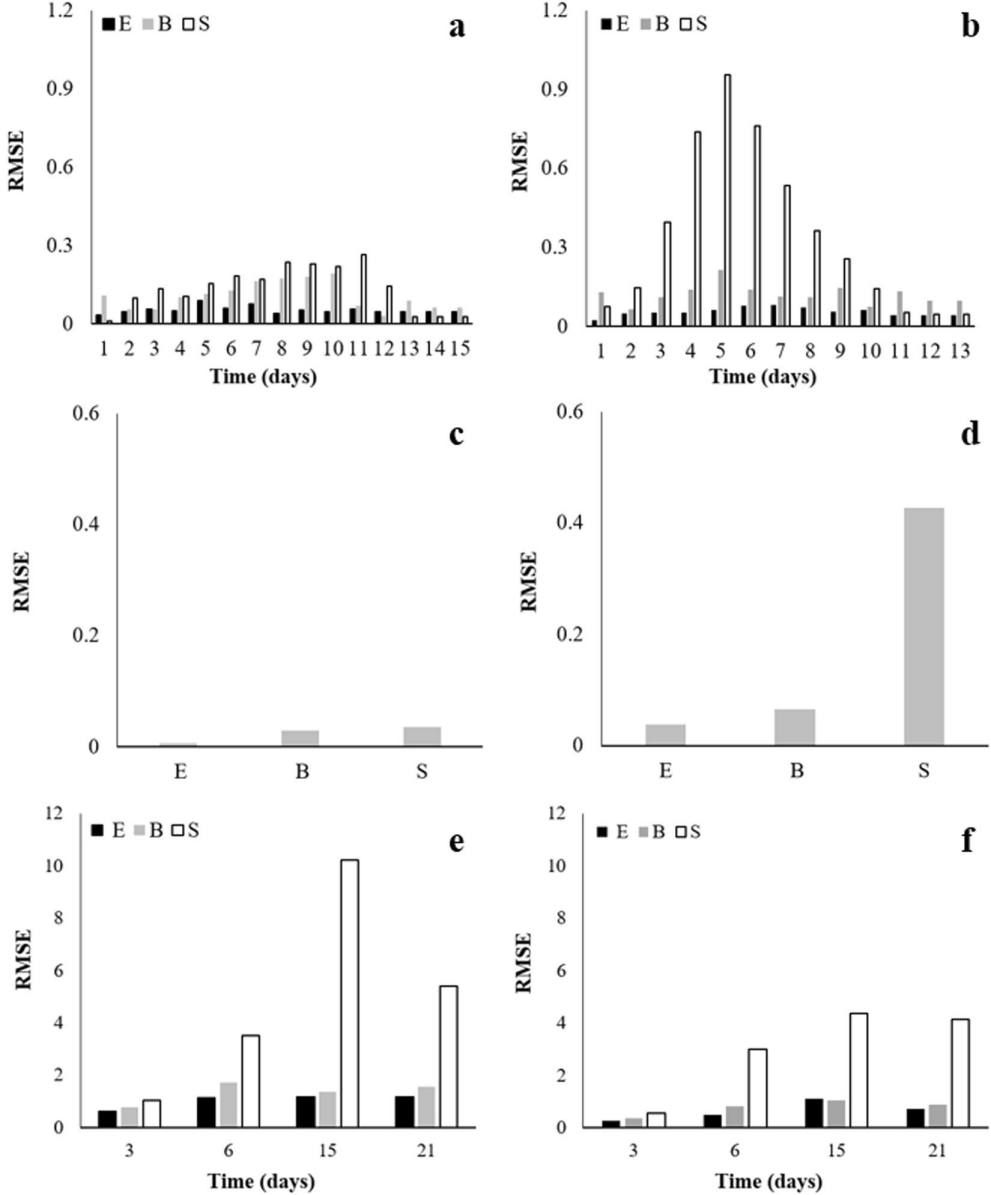
Experimental (E), Biological (B) and Strain (S) variabilities of daily growth of *A. carbonarius* (**a**) (15 days) and *A. niger* (**b**) (13 days); experimental (E), Biological (B) and Strain (S) variabilities of growth rate of *A. carbonarius* (**c**) and *A. niger* (**d**) and; experimental (E), Biological (B) and Strain (S) variabilities of OTA production by *A. carbonarius* (**e**) and *A. niger* (**f**) on days 3, 6, 15 and 21. Variability is expressed as root mean square error (RMSE).

When evaluating daily multiplication data, strain variability was greater than both biological and experimental variabilities for *A. niger*, on most days. As for *A. carbonarius*, the strain and biological variabilities did not differ, but were greater than the experimental variability, on most days by F-test. These results corroborate the variability found for strains of *Lactobacillus plantarum* and *Listeria monocytogenes*^16,42^. At the beginning and at the end of the evaluated period, *A. carbonarius* and *A. niger* appear to have greater biological variability (Fig. 2a,b). This may be related both to the initial phase of the experiment, in which the fungus is in the stage of adaptation and intense metabolic activity, and to the stationary phase (end of the experiment) in which the fungus may undergo changes in its mechanism, due to the scarcity of nutrients, which may not be reproducible in new experiments, even under similar conditions.

Strain variability is inherent to the microorganism, as it occurs even when they are submitted to the same conditions^43^. A higher coefficient of variation in the growth data of *A. carbonarius* strains in culture medium was also observed on the third day of incubation by Lappa, *et al*.^29^. Gougouli and Koutsoumanis^28^ observed a similar phenomenon in which, although the variation between the replicates was low in the optimal conditions, under conditions close to the growth limit, the experimental variability increased significantly. Baert, *et al*.^44^ also reported an increase in the variability of kinetic parameters of fungi under stress conditions; the highest variabilities were observed in the central period of the experiment. Typically, most filamentous fungal assays are conducted over 7 days; therefore, due to the great variability observed during this period, the use of a greater number of strains is important, so that predictions and results approach the reality.

By evaluating growth rate variability, we found that strain variability was higher than the biological and experimental (S>B=E) for *A. niger* species and, for *A. carbonarius*, the strain and biological variabilities were similar and greater than experimental variability (S=B>E) (Fig. 2c,d). When comparing the variabilities across species, greater variability of multiplication, both daily and in growth rate, is observed for *A. niger* (approximately ten times greater).

As far as OTA production is concerned, there is greater strain variability throughout the evaluated days, for both species, except on day 3 (E=B=S). However, *A. carbonarius* presented higher variability (Fig. 2e,f). On day 15, in which the highest levels of OTA were observed, greater variability was also observed. Therefore, in addition to the existing intra and inter-species variability, it is observed that such variability is dependent on time and response to be studied (growth/production of secondary metabolites). Lappa, *et al*.^29^ observed higher coefficients of variation between strains than between times of analysis of OTA production by strains of *A. carbonarius*, which reinforces the existence of inter-specificity across species. Although all strains were isolated from wine grapes cultivated in the tropical semi-arid region of Brazil, strains 10614 and 10625, the largest producers of OTA, were the only strains isolated from the region of Casa Nova-BA, Brazil. These data demonstrate that, in addition to geographic influence over the strain variability and variation of species isolated from grapes, there is also the influence of the microclimate of each region^2^. Moreover, genetic and phenotypic factors will also influence the production of secondary metabolites by strains within the same species^20,29^.

The use of a greater number of strains makes it possible to obtain information on the individual responses of the strains, which allows extreme conditions of growth and production of mycotoxin for a species to be known. Likewise, the difference in OTA production capacity and growth among strains indicates that the extrapolation of models obtained from data from only one individual strain may not be representative of most strains in a given species, leading to overestimation or underestimation of the predicted data^38^. Notwithstanding, it is important to emphasize that along with microbiological variability, there are also inherent variabilities to the process to which the food is submitted, although in some cases these are easier to control and the variability microbiology becomes the most determining factor in the final contamination^20^.

### Modified ochratoxin identification by HRMS

**Figure 3:**
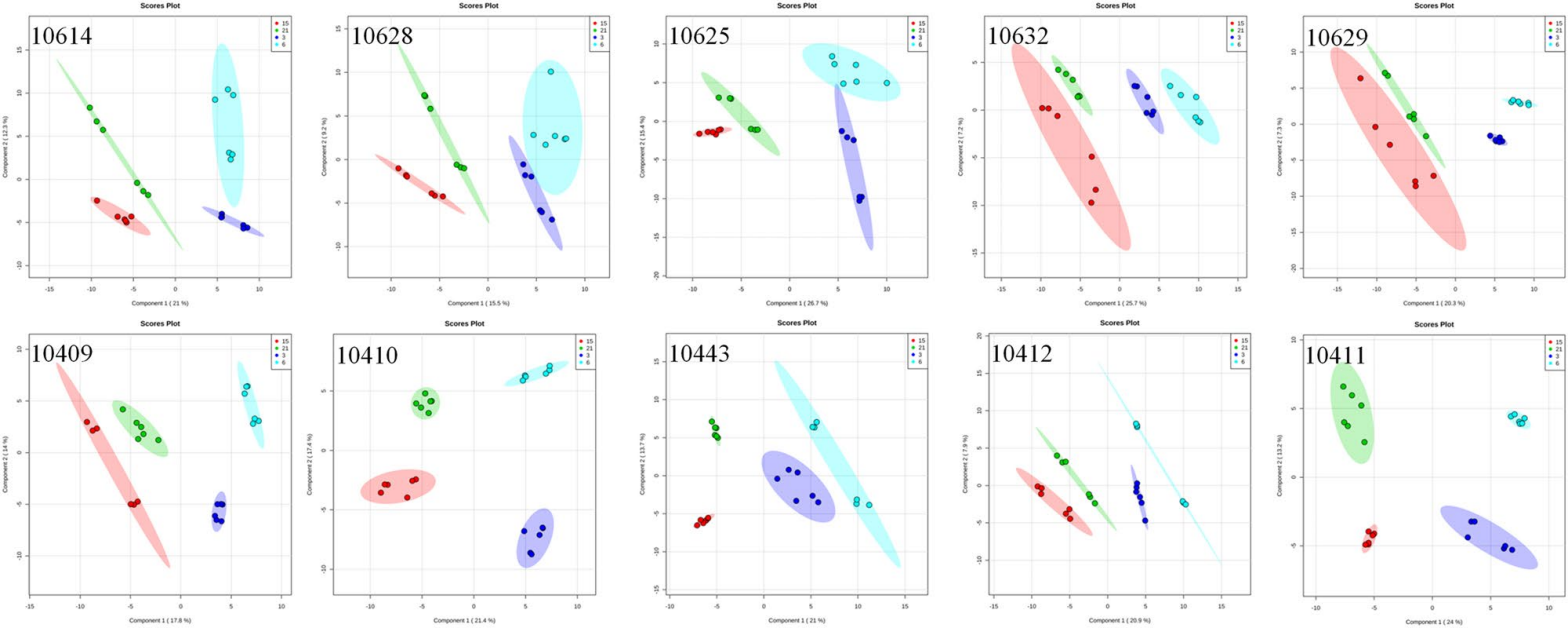
Scores plot of resulting from PLS-DA analysis for *A. carbonarius* (10614; 10628; 10625; 10632; 10629) and *A. niger* (10409; 10410; 10443; 10412; 10411) strains along 21 days of incubation in grape-based culture medium (Day 3: dark blue; Day 6: light blue; Day 15: red; Day 21: green).

The reduction of OTA observed between days 15 and 21 of incubation may be related to the formation of modified mycotoxins. To detect the presence of these metabolites, high resolution mass spectrometry and Partial Least Squares-Discriminant Analysis (PLS-DA) were used to assess the obtained mass spectral data. A discrimination of produced metabolites is observed, with the formation of 4 clusters, grouping samples with similar ion content, at times 3, 6, 15 and 21 days, for all studied strains (Fig. 3). Interestingly, biological variability is also observed within each cluster.

The loadings plot of the statistical model formed by features selected by PLS-DA indicated the 65 main candidate biomarkers of each strain over the assessed period (21 days). From this list of ions, it was possible to perform the search for modified mycotoxin. Among the targets sought, we identified only ethylamide-ochratoxin A ([M + Na]^+^: 453.1202) as a biomarker produced by strain 10443 (*A. niger*) on day 21. The model used by PLS-DA only shows the differences between the analyzed groups, and therefore ethylamide-ochratoxin A was the only molecule exclusive of one of the strains. It is possible, however, that other mycotoxins are also present, but have been produced by more than one strain at different times, which makes it impossible to identify these compounds as biomarkers. In addition, the reduction in OTA levels may also be related to a strong adsorption of mycotoxin by the matrix, making it impossible to extract it.

**Figure 4:**
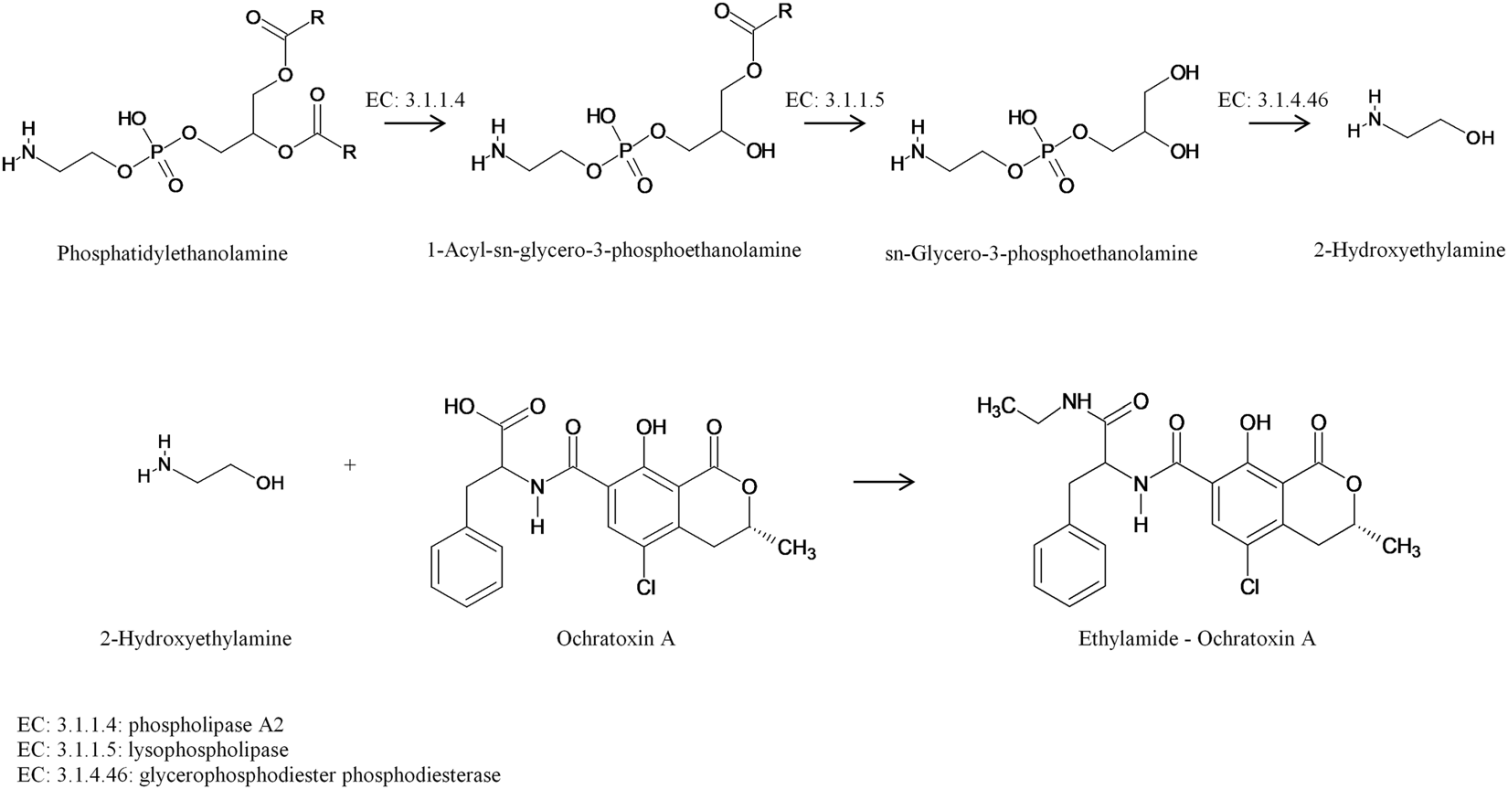
Glycerophospholipid metabolism of *A. niger* and proposal formation of ethylamide-OTA.

A series of isomeric candidate biomarkers within the same *m/z* range, 1-dodecanoyl-2-eicosapentaenoyl-glycero-3-phosphoethanolamine and/or 1-tetradecenoyl-2-octadecatetraenoyl-glycero-3-phosphoethanolamine and/or 1-octadecatetraenoyl-tetradecenoyl-glycero-3-phosphoethanolamine and/or 1-eicosapentaenoyl-2-dodecanoyl-glycero-3-phosphoethanolamine ([M + Na]^+^: 704.4248) was also identified among the metabolites produced by strain 10443 (*A. niger*) on day 15. Since this biomarker belongs to the phosphatidylethanolamine class, it may be a possible donor of the ethylamine group for the OTA molecule forming ethylamide-ochratoxin A. Such reaction may occur due to the enzymatic complex of the fungus capable of converting phosphatidylethanolamines into 2-hydroxyethylamine through the glycerophospholipid metabolism, reacting with OTA (Fig. 4).

The membrane of *A. niger* is mainly composed of phospholipids (85–90%), sterols (10–15%) and sphingolipids (1–2%). In the phospholipid fraction, the main representatives are: phosphatidylethanolamines, phosphatidylcholines, cardiolipins, and phosphatidic acids^45^. Phosphatidic acid, phosphatidylethanolamine, phosphatidylinositol, and phosphatidylserine were also detected as the major phospholipid species of the *Aspergillus* genus^46^. Under stress conditions such as thermal, osmotic, and oxidative shock, *A. niger* showed a reduction in the levels of phosphatidylethanolamines^45^. It is possible, therefore, that at the end of the incubation period, between 15 and 21 days, the metabolism of the fungus also changes due to the stress caused by nutrient shortages, reflecting the levels of phosphatidylethanolamine. These alterations may also be related to metabolic transformations and use of specific moieties of these lipids in the formation of modified mycotoxin. However, more studies are needed to elucidate which enzymes are associated with these reactions.

Some microorganisms are able to modify mycotoxins, mainly by degradation and conjugation reactions through their enzymatic complex^11^. A strain of *Saccharomyces cerevisiae* was shown to be able to convert mycotoxins, converting zearalenone to α-zearalenol, β-zearalenol, zearalenone-14-glucoside and zearalenone-16-glucoside^47^, and *Saccharomyces pastorianus* was also able to modify deoxynivalenol to deoxynivalenol-3-glucoside during fermentation^48^. The filamentous fungi belonging to the genus *Rhizopus* and *Thamnidium* were responsible for the metabolization of zearalenone to zearalenone glycosides, and *Fusarium* and *Aspergillus* species for zearalenone sulfates^49–52^.

Hydrolysis, hydroxylation, lactone opening, and conjugation reactions are the main biotransformation pathway of OTA^53^. The following biotransformation products: ochratoxin α, ochratoxin β, 4-R-OH-ochratoxin A, 4-R-OH-ochratoxin B, and 10-OH- ochratoxin A were detected in cultures of *Aspergillus ochraceus* incubated with OTA and ochratoxin B^54^. Ochratoxin α and L-β-phenylalanine were the metabolites generated by the degradation of OTA by *Aureobasidium pullulans*^55^.

The presence of these formed metabolites is another strong evidence that there may be underreporting of total mycotoxin levels, especially in fermented food products that are manufactured using microorganisms, as well as in products made from contaminated raw material^11,13^.

Our findings demonstrate the existence of both interspecies and intra-species variability, which confirms the need for selection of representative strains that can cover a wider range of conditions, considering their different kinetic behaviors. Such selection allows models to increasingly reflect reality and can be used in risk analysis, predicting product deterioration and/or the presence of mycotoxins. Whereas mycotoxins levels do not always correlate directly with growth, as demonstrated by the results presented here, the most effective control against OTA presence in food may be performed with fungus growth control. By avoiding the development of these species in food, mycotoxin production will not occur, thereby justifying the importance of modeling the growth of potentially mycotoxigenic fungi, and not only the factors that will influence mycotoxin production evidenced in secondary models. The description of strain growth over time is the basis for adopting preventive practices, from the field to the consumer, assisting in food quality and safety^21,56^. Moreover, the use of quantitative modeling is a crucial tool in Hazard Analysis and Critical Control Points, and also helps determining the maximum tolerable limits of mycotoxins.

In addition, the reduction of OTA levels produced by *A. carbonarius* and *A. niger* in the final incubation period, may be related to the formation of modified mycotoxins by the fungus itself or by adsorption of the compound in the matrix. In both cases, the results demonstrate the possibility of underreporting of the total levels of mycotoxins present and a potential health risk. Furthermore, modified mycotoxins may be reconverted into the parent mycotoxin, either by the industrial process itself, or in the digestive system after ingestion of the contaminated food, recovering the toxicity potential responsible for health effects in humans and animals^13,51^.

## Material and Methods

### *Aspergillus carbonarius* and *A. niger* strains

Five strains of *Aspergillus carbonarius* and five of *A. niger*, isolated from grapes, were obtained from the Culture Collection of the Department of Food Science/CCDCA-UFLA. *A. niger* strains: 10409, 1410, 10443, 10412 and 10411 were isolated from grapes collected in Petrolina - PE - Brazil. *A. carbonarius* strains: 10628, 10632 and 10629 were isolated from grapes collected in Lagoa Grande - PE – Brazil, and strains: 10614 and 10625 from Casa Nova - BA - Brazil. Strains were previously characterized as potentially ochratoxigenic by the Plug Agar method in thin layer chromatography, according to Filtenborg and Frisvad (1980)^57^.

### Preparation of the conidial suspensions

Conidial suspensions of each strain were individually prepared and had their concentration standardized at 10^6^ conidia/mL according to Wigmann, *et al*.^58^. *A. carbonarius* and *A. niger* strains were inoculated in MEA- Malt Extract Agar medium (Acumedia) (Malt Extract: 20.0 g, Peptone: 1.0 g, Glucose: 30.0 g, Agar: 20 g, Distilled Water: 1 L) and incubated at 25 °C for 7 days. Conidia were collected by scraping the mycelium from each plate with sterile distilled water and 0.1% Tween 80 (Labsynth). Subsequently, they were filtered and then centrifuged at 11962.6 × g three consecutive times for 15 minutes at 5 °C (Sorvall Legend XTR, Thermo Scientific, Hampton, USA). The final concentration of conidia in each fungal suspension was determined in Neubauer chamber (Sigma-Aldrich).

### Preparation of the grape-based culture medium and inoculation of strains

The experimental, biological and strain variabilities between strains of multiplication and OTA production were evaluated in a culture medium based on Syrah grapes, according to Passamani, *et al*.^31^. The medium was prepared by adding 175 mL of juice obtained from crushed grapes in 825 mL of distilled water and 20 g of agar (Inlab). A 10 μL aliquot of the conidial suspension of each strain was inoculated individually, and incubated at 25 °C, over 21 days. The control plate was inoculated with 10 μL of sterile distilled water without spore suspension.

### Growth evaluation and mathematical modeling

The mycelial radial growth of the strains was evaluated by daily measurement (one measure/day) of the colony diameter (mm) in two perpendicular directions during 21 days, using a digital caliper with a 0.01 mm resolution (Digimess -100.175BL, Brazil). The Baranyi and Roberts model^59^ (Equations 1 and 2) were fitted to data on colony diameter as a function of time, using DMFit software, an Excel add-in. The multiplication rate (μ_max_, mm.day^−1^) and lag phase time (λ, days) were estimated after model fitted.

1 $$D(t)={\mu }_{{\rm{\max }}}A-\,\mathrm{ln}[1+\frac{\exp \,({\mu }_{{\rm{\max }}}A)-1}{\exp ({D}_{{\rm{\max }}})}]$$

2 $$A=t+(\frac{1}{{\mu }_{{\rm{\max }}}})\mathrm{ln}[\exp (-{\mu }_{{\rm{\max }}}\,t)+\exp (-{\mu }_{{\rm{\max }}}\,\lambda )-\exp (-{\mu }_{{\rm{\max }}}\,t-{\mu }_{{\rm{\max }}}\,\lambda )]$$

where: D(t) is the colony diameter (mm) as a function of time, µ_max_ is the maximum multiplication rate (mm.day^−1^), λ is the time obtained by the intersection (days), and D_max_ is the maximum diameter of colony.

### Determination of OTA production

#### Sample preparation and extraction

In order to determine the levels of OTA produced by the strains, three pieces of the grape-based medium were removed from the internal, middle and external areas of each colony during 21 days of incubation at 25 °C (day 3, 6, 15 and 21). The reagents used were all of high purity or HPLC grade (99.9%). For extraction, 1 mL of methanol (Sigma-Aldrich) was added, followed by vortex homogenization for 5 seconds and incubation at room temperature for 60 minutes. The extracts were filtered in PVDF - Polyvinylidene Fluoride (0.22 μm) filter units (Millipore), according to the methodology proposed by Bragulat, Abarca, and Cabañes^60^ and then submitted for quantification. The standard curve was prepared using a stock solution previously prepared by dissolving the commercial OTA standard (Sigma-Aldrich) in methanol (1 μg/mL). Subsequently, standard solutions with concentrations of 3.75; 15.0; 30.0; 105.0 and 135.0 ng/mL were prepared by dilution. For the recovery assay, the grape-based culture medium was spiked at three levels with concentrations equal to 1.0 μg/g; 3.0 μg/g and 6.0 μg/g, in triplicates. The results of the recovery trials were 98.32% (±12.29), 97.23% (±7.07), and 97.47% % (±7.82), respectively.

#### OTA quantification by HPLC

OTA quantification was performed on an Agilent Techonlogies 1290 infinity HPLC with a DAD (diode array) detector at the wavelength of 330 nm. The Agilent-Zorbax Eclipse XDB-C18 column (4.6 × 250 mm, 5 μm) was used, with a column temperature of 25 °C, flow of 0.5 mL.min^−1^ and injection volume of 20 μL. Elution was performed in an isocratic system of acetonitrile: methanol: aqueous acetic acid (35: 35: 29: 1) (J. T. Baker). The mean retention time obtained for OTA was 4.7 ± 0.1 min. OTA quantification was performed by building an analytical curve obtained by linear regression (y = 4987.8× + 86.735), with determination coefficient (R^2^) of 0.997. The limits of detection (LoD) and quantification (LoQ) were 0.001 and 0.004 μg/g, respectively. All samples were analyzed in duplicates, and standard OTA solutions were injected in triplicates.

### *A. niger* and *A. carbonarius* variability of multiplication and OTA production

Experimental variability was quantified by conducting the experiment in duplicate at the same time using the same conidial suspension. To quantify biological variability, the experiment was also replicated two more times on different days using fresh suspensions. This procedure resulted in six colony diameter values (mm) and OTA concentrations (μg/g). Subsequently, the experimental variability (E), biological (B), and strains (S) indexes, in relation to the multiplication and production of OTA, were determined^16^. (Equation 3: Experimental variability; 4: Biological variability; 5: Strain variability):

3 $${MSE}=\frac{RSS}{DF}=\frac{{\sum }_{S=1}^{5}{\sum }_{B=1}^{3}{\sum }_{E=1}^{2}{({X}_{{EBS}}-{X}_{BS})}^{2}}{n-{p}}$$

4 $$MSE=\frac{RSS}{DF}=\frac{{\sum }_{S=1}^{5}{\sum }_{B=1}^{3}{({X}_{BS}-{X}_{S})}^{2}}{n-p}$$

5 $$MSE=\frac{{RSS}}{DF}=\frac{{\sum }_{S{=}1}^{5}{({{X}}_{{s}}{-}X)}^{2}}{n-p}$$

Where: MSE is the mean square error calculated from the sum of the squares of the residuals (RSS) divided by the degree of freedom (DF); X_EBS_ is the diameter (mm) or concentration of mycotoxin (μg/g) obtained from each replicate of the duplicate experiment performed at the same time “E”, biological reproducibility “B”, and strain “S” (E = 1, 2; B = 1.2, S = 1, 2, 3, 4, 5); X_BS_ is the mean of X_EBS_ for the “S” strain obtained on the same day; X_S_ is the mean of X_BS_ of three experiments performed on different days for strain “S”, X is the mean of X_S_ for the five strains; (n-p) is the number of data minus the number of parameters. F-test was used to compare the experimental, biological and strain variability with an alpha of 0.05. The variabilities were expressed as root mean square error (RMSE).

### Modified ochratoxin identification by HRMS

For search and identification of OTA derivatives: ochratoxin β (222.0528 g/mol), α-ochratoxin (256.0139 g/mol), α-ochratoxin amide (255.0298 g/mol), 14-decarboxy-ochratoxin A (359.0924 g/mol), ochratoxin B (369.1212 g/mol), ochratoxin B methyl ester (383.1369 g/mol), ochratoxin B ethyl ester (397.1525 g/mol), ochratoxin A (403.0823 g/mol), 4-hydroxyochratoxin A (419.0772 g/mol), ethylamide ochratoxin A (430.1296 g/mol), ochratoxin A glucose ester (565.1351 g/mol), (22 → 6′) ochratoxin A-methyl-α-D-glucopyranoside ester (579.1507 g/mol), ochratoxin A cellobiose ester (727.1879g/mol), ochratoxin A quinone (383.1005 g/mol), ochratoxin A hydroquinone (385.1162 g/mol), ochratoxin C (431.1136 g/mol); 10 μL of the obtained extract (item 2.5.1) were diluted in 490 μL of methanol and homogenized under vortex for 30 seconds. Then, 0.1% formic acid was added and the sample was directly injected. An ESI-LTQ-XL Orbitrap Discovery (Thermo Scientific, Bremen, Germany) mass spectrometer with a nominal resolution of 30,000 (FWHM) was used to acquire the data in the survey scan mode. The parameters used were: flow rate at 10 μL.min^−1^, capillary temperature at 280 °C, spray current at 5 kV, and sheath gas at 5 arbitrary units. Data were obtained in the positive mode using mass range of 250–750 *m/z* in triplicate.

### Statistical analyses

Analysis of variance (ANOVA), with *a posteriori* Tukey test, was used to evaluate the difference in growth parameters and OTA levels by *Aspergillus carbonarius* and *A. niger* strains in a grape-based medium. To assist in the identification of OTA derivatives, the multivariate regression method, Partial Least Squares-Discriminant Analysis (PLS-DA) was used. The analyses were performed through the online platform MetaboAnalyst 3.0^61,62^, using interquartile range for data filtering, and quantile normalization. The Lipid MAPS online database (University of California, San Diego, CA) and METLIN (Scripps Center for Metabolomics, La Jolla, CA), as well as bibliographic references were consulted to identify compounds of interest through their exact mass, with a maximum error of 2 ppm.
