# Author Correction: A quantitative study on growth variability and production of ochratoxin A and its derivatives by A. *carbonarius* and A. *niger* in grape-based medium

**DOI:** 10.1038/s41598-019-42136-7

**Published:** 2019-04-26

**Authors:** Luísa Freire, Tatiane M. Guerreiro, Arthur K. R. Pia, Estela O. Lima, Diogo N. Oliveira, Carlos F. O. R. Melo, Rodrigo R. Catharino, Anderson S. Sant’Ana

**Affiliations:** 1 Department of Food Science, Faculty of Food Engineering, University of Campinas, Campinas, SP Brazil; 2 Innovare Biomarkers Laboratory, Faculty of Pharmaceutical Sciences, University of Campinas, Campinas, SP Brazil

Correction to: *Scientific Reports* 10.1038/s41598-018-32907-z, published online 01 October 2018

The Acknowledgements section in this Article is incomplete.

“L. Freire acknowledges the financial support of São Paulo Research Foundation: Grant # 2016/21041-5, São Paulo Research Foundation (FAPESP). The authors acknowledge the Culture Collection of the Department of Food Science/CCDCA-UFLA for the donation of fungi strains used in this study. The authors are also thankful to Conselho Nacional de Desenvolvimento Cientifico e Tecnológico (CNPq) (Grant # 302763/2014-7). This study was financed in part by the Coordenação de Aperfeiçoamento de Pessoal de Nível Superior - Brasil (CAPES) - Finance Code 001”.

should read:

“L. Freire acknowledges the financial support of São Paulo Research Foundation: Grant # 2016/21041-5, São Paulo Research Foundation (FAPESP). The authors acknowledge the Culture Collection of the Department of Food Science/CCDCA-UFLA for the donation of fungi strains used in this study. The authors are also thankful to Conselho Nacional de Desenvolvimento Cientifico e Tecnológico (CNPq) (Grant # 302763/2014-7). This study was financed in part by the Coordenação de Aperfeiçoamento de Pessoal de Nível Superior - Brasil (CAPES) - Finance Code 001. ASS acknowledges the support of FAPESP (grant #2018/19341-6)”.

